# Epigenetic regulation of histone H3 in the process of hepatocellular tumorigenesis

**DOI:** 10.1042/BSR20191815

**Published:** 2019-08-02

**Authors:** Dan Li, Zhenguo Zeng

**Affiliations:** 1 Department of Critical Care Medicine, the First Affiliated Hospital of Nanchang University, Nanchang, Jiangxi 330006, China; 2 Department of Pharmacology, University of Illinois College of Medicine, Chicago, IL, U.S.A.

**Keywords:** DNA synthesis and repair, epigenetic regulation, Hepatocellular carcinoma, histone H3, inflammation, tumorigenesis

## Abstract

Better understanding of epigenetic regulation of hepatocellular carcinoma (HCC) will help us to cure this most common malignant liver cancer worldwide. The underlying mechanisms of HCC tumorigenesis are genomic aberrations regulated by genetic and epigenetic modifications. Histone H3 lysine modifications regulate histone structure and modulate transcriptional factor binding with target gene promoters. Targetting genes include VASH2, fatty acids synthase, RIZ1, FBP1, MPP1/3, YAP, which affect tumorigenesis, metabolisms, angiogenesis, and metastasis. Signal pathway studies demonstrate that the HGF-MET-MLL axis, phosphatase and tensin homolog (PTEN)-PI3K-Akt axis; WNT-β-catenin signal pathway is involved in histone H3 modification. A variety of factors such as virus infection, reactive oxygen species, food-borne toxins, irradiation, or non-coding RNA cause hepatocellular DNA damage or modification. Dysfunctional DNA repair mechanisms, including those at the epigenetic level are also major causes of HCC tumorigenesis. The development of therapies based on epigenetic regulatory mechanisms has great potential to advance the care of HCC patients in the future.

## Introduction

The well-established definition of an epigenetic modification is a ‘stably heritable phenotype resulting from changes in a chromosome without alterations in DNA sequence’ [1]. However, accumulating evidence now suggests that epigenetic modification encompasses much more than this. The latest definition by NIH states that ‘epigenetic refers to both heritable changes in gene activity and expression (in the progeny of cells or of individuals) and also stable, long-term alterations in the transcriptional potential of a cells that are not necessarily heritable’ [2]. Epigenetic changes modify the activation of certain genes, but not the genetic code of sequences of DNA. These changes include DNA methylation or demethylation and histone amino acids modification [3]. The most common histone modifications include histone lysine or arginine methylation, acetylation, phosphorylation statuses changes. Histone modifications regulate histone structure and control transcriptional factors binding with target gene promoter or regulatory DNA elementary region. [4]. Epigenetic modification plays a critical role in gene expression, and is involved in almost all cellular processes, such as metabolism, growth, differentiation, and tumorigenesis [5,6].

Hepatocellular carcinoma (HCC) is the most common malignant liver cancer and it causes 662,000 deaths every year worldwide [7]. Nearly half of these cases have happened in China. In underdeveloped countries, HCC is dominantly caused by infectious disease, such as viral hepatitis B or C, or by food toxin such as aflatoxin [8]. On the other hand, in the developed countries, the majority of HCC cases are caused by alcohol or genetic reasons such as hemochromatosis and α1-antitrypsin deficiency [9]. HCC development is divided into the early stage and the late stage. In the early stage, inflammatory responses, such as cytokine secretion or proliferation, play a critical role in the eventual development of HCC. In the late stage, chronic inflammatory response, such as cirrhosis and necrosis also play important roles in HCC. Interestingly, epigenetic regulation is involved in HCC development in both the early and late stages [10]. In this review, the histone H3 modifications and regulatory signal pathway in the process of HCC development have been systematically reviewed.

## Histone H3 modification

### Histone 3 lysine acetylation

Histone H3 modifications have been intensively investigated in various cancer models, including HCC. There are several major histone H3 modifications during HCC development: histone methylation and acetylation, histone phosphorylation and ubiquitination. Amongst all the histone modifications, histone methylation and acetylation have been intensively investigated as playing a role in HCC development.

**Figure 1:**
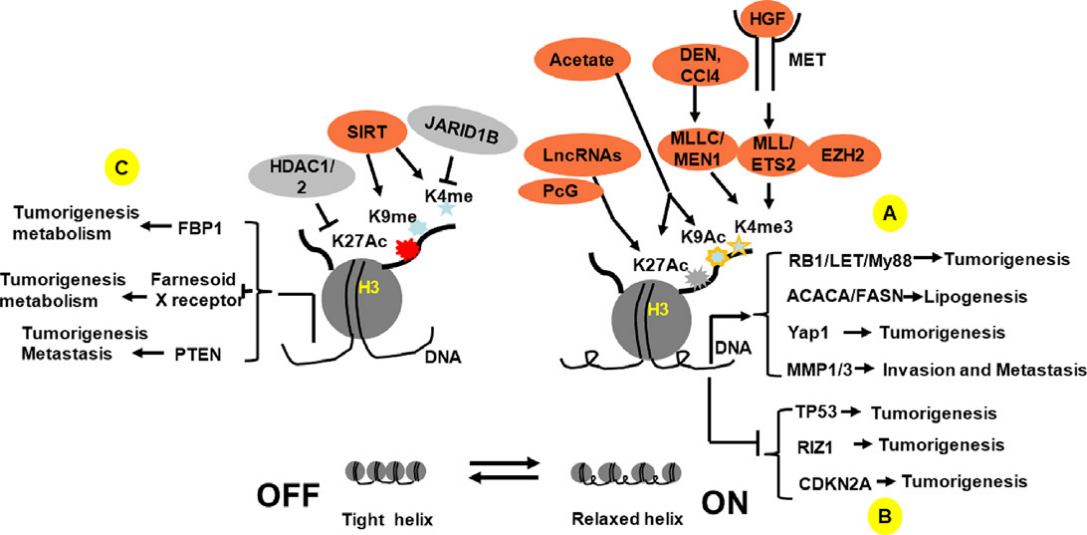
Schematic description of histone H3 modification-mediated target genes regulation and relative signal pathways **(A)** By directly turning on oncogene expression (RB1, My88) or targetting the transcriptional factors or enhancers (Yap1, FOXAs), H3 histone modifications enhance oncogene expression and promote tumorigenesis; **(B)** By inactivation of the tumor suppressor gene (RIZ1, EZH2) or relative transcriptional factors, H3 histone modifications indirectly enhance oncogene (P53) or relative gene (P16, CDKN2A, TP53) expression and promote tumorigenesis; **(C)** By modifying the transcriptional factor binding with target gene promoter, histone H3 modifications change HCC behavior (VASH2, MMP1/3, PTEN) or metabolism (FASN, FBP1, FXR) properties.

There are abnormally high H3K4 trimethylation and H3 acetylation, as well as a lower H3K27 trimethylation at putative vasohibin 2 (VASH2) promoter in HCC, which results in an abnormally high expression of VASH2 in HCC. In contrast, silence of VASH2 is known to inhibit the HCC proliferation while enhancing apoptosis. Moreover, the secreted VASH2 from HCC also enhances human umbilical vein endothelial cells growth and proliferation, indicating that VASH2 may functions as growth factor [11]. Sterol regulatory element-binding transcription factor 1c (SREBP-1c) and carbohydrate responsive-element binding protein (ChREBP) are major regulatory factors for fatty acids synthase (FASN). In normal tissue, insulin induces these transcriptional factors binding with sterol regulatory elements (SRE) or carbohydrate responsive-elements (ChORE) of the FASN promoter and induces FASN expression. However, in HCC, hyperacetylation of histoneH3 an H4 impairs SREBP-1c-SRE and ChREBP-ChORE binding on FASN promoter and HCC becomes insulin resistant [12] (Figure 1).

Human HCC cell in culture display a nucleosome density that is relatively lower than control, which accompanies histone H3 acetylation at lysine 9 (K9), regardless of their transcriptional activation status. The present study indicates that K9 acetylation may play an important role in nucleosome relaxing and in tumorigenesis initiation [13]. The retinoblastoma-interacting zinc finger gene RIZ1 is inactivated in many cancers. All 323 HCC tissues with promoter methylation showed complete loss of the RIZ1 protein, whereas RIZ1 protein was present in all the corresponding non-cancerous tissues. Neither 5-Aza-2-deoxycitidine (5-Aza-dC) nor trichostatin A (TSA) reversed promoter methylation, but both did restore RIZ1 mRNA. This indicates that 5-Aza-dC or TSA regulates RIZ1 through the histone acetylation modification mechanisms. Further studies show that relative lower levels of H3K9 acetylation (H3K9 methylation/acetylation ratio increased in HCC) contributes to silencing RIZ1 in HCC [14]. Acetylation and tri-methylation of H3K27 concurred in a subset of HCC, which may be connected a more aggressive HCC tumor phenotype. A clinical HCC samples assay showed that HCC tissues have significantly higher H3K27ac and H3K27me3 scores compared with the background liver. Concurrent acetylation and methylation at H3K27 in HCC cells also associate with P53 abnormalities [15]. In HCC patients, fructose-1,6-bisphoshatase (FBP1) is down-regulated and associated with a poor prognosis. Treatment of HCC cells with histone deacetylase (HDACs) inhibitor or knockdown of HDAC1 and/or HDAC2 restored FBP1 expression and inhibited HCC cells growth. HDAC-mediated suppression of FBP1 expression correlates with a decrease of histone H3 lysine 27 acetylation (H3K27Ac) in FBP1 enhancer [16]. A HDAC inhibitor, suberoylanilide hydroxaminic acid (SAHA) analog, significantly inhibited cellular proliferation and induced apoptosis in HCC cell lines HepG2 and SK-HEP-1. These effects are associated with hyperacetylation of histone H3 at various positions on the lysine residue [17].

### Histone 3 lysine methylation

The connection between HCC development and histone H3 lysine methylation has also been widely investigated. Early studies show that a higher level of H3K4 trimethylation is associated with a poor prognosis of HCC [18], and HCC has lower H3K4 dimethylation, which may be associated with a higher level of H3K4 methylating enzyme complex, Ash2. In contrast, HCC lacks demethylating enzymes (LSD1) compared with other kinds of cancer cell lines [19]. Mixed-lineage leukemia (MLL), a histone methyltransferase and an epigenetic regulator, causes trimethylation of H3K4. H3K4me3 is necessary for MLL-ETS2 complex to occupy the promoter of MMP1/MMP3 resulting in activation of MMP1/MMP3 expression. These results indicate that MLL-mediated H3K4 trimethylation is necessary for HGF induced-HCC proliferation and metastasis [20]. Menin is a scaffold protein encoded by the multiple endocrine neoplasia type 1 (MEN1) gene. Heterozygous ablation of MEN1 in female mice reduces chemical carcinogen-induced liver carcinogenesis and represses the activation of the inflammation pathway (Figure 1). In HCC tissue CHIP assays, the binding of menin and accumulation of H3K4me3 at the YAP1 promoter was markedly increased in HCC species compared with adjacent tissue [21]. Agrin, a 210kDa proteoglycan which is highly expressed in HCC, can activate YAP gene and cause HCC metastasis and invasion [22]. Signal pathway analysis shows that Agrin regulates actin stress fibers. And further activates YAP gene by modulating LRP4-Musk signal pathway; meanwhile, Agrin turns off hippo tumor suppressive gene by activation of FAK-ILK-PAK1 signal pathway [23]. While not the focus of these studies, epigenetic regulation may well play a role in this process. Hepatitis B virus X protein induces H3K9me3 on the p16 promoter via the decrease of demethylase JMjd2B expression and thus promotes the repression of p16 gene expression to enhance hepatocarcinogenesis in a cellular model [24].

Enrichment of H3K27 trimethylation, independent of H3K9 dimethylation, trimethylation, and DNA methylation, was an early event in the silence of p16 (INK4a) during the tumor development [25]. Chronic hepatitis C (CHC) patient samples and H_2_O_2_ treatment of the hepatocellular cell lines studies show that histone is modified from an active chromatin (trimethyl-H3K4 and acetylated-H4K16 dominant) to a repressive chromatin (trimethyl-H3K27 dominant) status. These results indicate that oxidative stress alters the chromatin status, which leads to abnormal methylation of TSGs and contributes to hepatocarcinogenesis in CHC patients [26]. Concurrent activation of acetylation and tri-methylation of H3K27 is associated with an aggressive HCC phenotype [15]. By H3K27-dependent and H3K27-independent mechanism, polycomb group genes (PcGs) effectively block the aggressive signature of liver cancer cells *in vitro* and *in vivo* [27].

The relationship of acetylation and methylation on histone lysines is sophisticatedly and precisely regulated by different enzymes. Histone H3 acetylation and methylation of several lysine residues could happen simultaneously. Moreover, other targetting amino acids of histone H3, such as H3K18 and H3K56 or arginine, were also modified as HCC develops. However, there are just a few reports describing their role in the process of tumorigenesis of HCC [28,29].

## Long non-coding RNA is also involved in the HCC tumorigenesis by regulation of histone modification

Long non-coding RNA (lncRNA) is a member of non-coding RNA family, which is characterized as longer than 200 base pairs. unlike miRNA and circRNA, lncRNA can form secondary structure and can be post-translationally processed through splicing or 5′capping. Accumulating evidence shows that lncRNA are involved in inflammatory process, especially in chronic inflammatory process. The major targetting signal pathways of lncRNAs include NF-κB pathway, JAK-STAT pathway, and MAPK pathway. Aberrant regulation of these signal pathways causes cell inflammatory response, cell apoptosis, or neurodegerative diseases [30]. Moreover, lncRNA is also involved in tumorigenesis of HCC which accompanies the inflammatory response. The tumor suppressor gene, retinoblastoma (RB1) was aberrantly lower in HCC. A bidirectional transcriptional lncRNA (Linc00441) had a relatively higher level in HCC. Linc00441 causes hypermethylation of RB1 and inhibits RB1 expression by recruiting DNMT3A. Moreover, Linc00441 promoter CHIP studies show that there is a higher level of H3K27ac and a lower level of H3K4me2 in HCC. Bidirectional transcriptional of Linc00441 and RB1 via H3K27 modification-dependence promoted HCC [31]. LncRNA-LET, a recently identified IncRNA has been shown to be a tumor suppressor for HCC. LncRNA-LET is repressed by EZH2-mediated H3K27 histone methylation on the LET promoter [32]. Another lncRNA, lnc-Myd88 has aberrantly high level in HCC. By enhancing H3K27ac at the promoter of Myd88, Lnc-Myd88 increased Myd88 expression in HCC (Figure 1). Myd88 in turn activated NF-κB and PI3K-Akt signal pathway, and those enhanced HCC growth and metastasis [33].

## Signal pathways

MLL is a transcriptional coactivator playing a central role of HCC early development. MLL binds with transcription factors and drives target gene expression. MLL also plays critical role in epigenetic regulation of cell cycle and self-renewal of stem cells [34]. In cellular and animal models of HCC, HGF signals through its cognate receptor, MET, which drives MLL–ETS complex binding with MMP1 and MMP3 promoter. At same time, MLL induces H3K4 trimethylation. Both mechanisms activated MMP1 and MMP3 expression in HCC and caused invasion and metastasis [20].

JARID1B is a member of the family JmjC domain-containing proteins that removes the methyl group from methylated lysine 4 on histone H3 (H3K4). Comparing with normal liver tissue, HCC has significantly higher mRNA and protein level of JARID1B which is associated with metastasis and poor survival rate. Silencing of JARID1B in HCC cell lines, such as HepG2, SNU423, or SK-Hep1, inhibited their proliferation, migration, or invasion. *In vivo* experiments have shown that exogenous implanting HepG2 with overexpressing JARID1B results in larger tumor size compared with control. Signal pathway analysis showed that higher level of JARID1B in HCC inhibited PTEN expression by demethylating H3K4me3 associated PI3K-AKT signal pathway activation, which eventually promotes metastatic behaviors of HCC [35]. A recent study showed that basil polysaccharide suppressed HCC migration and invasion in a rat model. By ligation of hepatic artery, the intratumoral hypoxia caused HCC metastasis. Basil polysaccharide administration inhibited this metastasis by down-regulation of JARID1B accompanying H3K9me2 demethylation [36] (Figure 1).

Sirtuin 1 (SIRT1) is involved in many hepatocellular metabolisms, such as lipogenesis, protein synthesis, glucogenesis, and bile acid (BA) homeostasis. SIRT1 has been found to be aberrantly high in HCC compared with health control. Signal pathway assays showed that higher level of SIRT1in HCC up-regulates AMPK. AMPK negatively regulates mTORC1 and p70S6K signal axis accompanying histone H3 K4 and K9 trimethylation. Both of them result in farnesoid X receptor (FXR) expression inhibition. Moreover, comparing with control, hepatectomy of the overexpression SIRT1 mice increased mice mortality, decreased proliferation of hepatocytes which was accompanied by decreased expression and activity of farnesoid X receptor (FXR). The aberrant expression and deacetylation of farnesoid X receptor (FXR) induced by higher level of SIRT1 resulted in hepatocytocellular abnormality, such as BA metabolic dysfunction, hepatocellular tumorigenesis [37]. By epigenetic regulation, SIRT1 also up-regulates telomerase (TERT) expression and activity in HCC. In the culture HCC models, such as SK-HEP, SNU-423, or Hep3B, silencing of SIRT1 resulted in inhibition of TERT expression. Interestingly, silence of SIRT1 did not change promoter activity or mRNA stability of TERT in HCC. However, silencing of SIRT1 is associated with substantial induction of H3K9 acetylation and reduction of trimethylation at the TERT gene. Although the detail mechanism, such as the signal pathway involved remains unclear, the study indicated that epigenetic regulation may play a central role in tumorigenesis and proliferation of HCC [38]. Exposure of HepG2 cells to trichostatin A (TSA) inhibits cells proliferation and increase cell percentages in G0/G1 and G2/M phases while decreasing the percentage in S phase. TSA significantly induces β-catenin, H3K9 and Bax gene induction and significantly inhibits cyclin D1, HDAC1/3 expression in HepG2 cells. This indicates that TSA-mediated HDAC inhibition causes histone hyperacetylation and activation of Wnt-β-catenin signal pathway. These effects cause inhibition of HCC proliferation, cell cycle arrest and apoptosis [39].

Polycomb group (PcG) proteins are highly conserved epigenetic gene silencers that play important roles in the maintenance of cell fates. On HCC model studies showed that reduced one component of polycomb repressive complex 2(PRC2), enhancer of zeste homolog 2 (EZH2), effectively decreases invasion, and proliferation of liver cancer cell *in vitro* and *in vivo*. EZH2 negatively regulates TP53, a tumor suppressor in HCC. But EZH2 positively regulates E2F1, NOTCH2 expression in HCC. CHIP assay showed that EZH2 and H3K27me3 coincidently occupied the promoter of cyclin-dependent kinase inhibitor (CDKN2A), but not the TP 53 promoter. The results indicate these genes are regulated by EZH2 in a H3K27me3-dependent or H3K27me3 independent way. Moreover, survival rate of the patients expressing EZH2 and menin was significantly lower than the patients not expressing EZH2 or menin. Further, inhibition of H3K27me3 alone or inhibition of H3K4me3 together effectively blocked the aggressive phenotype of HCC cells [27,40] (Table 1).

**Table 1:** The target genes of histone modification and relative clinical consequence

Histone modification	Cause	Target gene/Signal	Consequence
H3K9 trimethylation +	Alcohol	VASH2 ↑	Proliferation +
H3K27 trimetylation -			Apoptosis -
H3K4 trimethylation +	Non-alcohol	FASN ↓	Insulin resistance
H3 acetylation +			
H3K9 acethylation +	HVB	RIZ1 ↓	Proliferation +
H3K9 trimetylation +			
H3K27 acethylation +	Alcohol	P53 ↓	Aggression +
H3K27 trimetylation +			Invasion +
H3K27 acethylation +	Alcohol	FBP1 ↓	Metabolism and poor prognosis
H3K4 trimethylation +	MLL	HGF ↑	Proliferation +
			Metastasis -
H3K4 trimethylation +	Chemicals	YAP1 ↑	Proliferation +
			Inflammation -
H3K9 trimethylation +	HVB	P16 ↓	Proliferation +
H3K4 trimethylation +	CHC	TSG ↓	Proliferation +
H3K16 acethylation +			
H3K4 dimethylation -	Linc00441	RB1 ↓	Proliferation +
H3K27 acetylation +			Apoptosis -
H3K27 acetylation +	Lnc-Myd88	NF-kB ↑	Proliferation +
			Metastasis +

## The importance of histone modification for DNA repairing during the HCC development

### Aberrant histone modification results HCC tumorigenesis and development

The DNA damages in hepatocyte include DNA deletion, mutation, or modification. Damage may be repaired by various mechanisms at the genetic or epigenetic level. However, unrepaired DNA will be translated into aberrant protein. Accumulating evidence shows that the leader causes of HCC tumorigenesis are alcohol, hepatitis virus B/C, food-borne toxins, chemicals as well as oxidative stress [26,41]. HBV DNA inserts into host chromosomes and initializes hepatocellular carcinogenesis. During this process, HBV also causes host DNA methylation by inhibition of methyltransferase [42]. HCV-mediated HCC tumorigenesis by targetting the enhancers of FOXAs, HNF4A. These enhancers are regulated by enhancement of H3K27me3 in hepatocytes [43].

Exposure to chemicals is also an important cause for HCC tumorigenesis and metastasis. Diethylnitrosamine/tetrachloride (CCl_4_) induced HCC tumorigenesis by decrease of H3K9me3, genomic DNA demethylation, and down-regulation of RIZ1, a histone lysine methyltransferase tumor suppressor gene [44]. In a diethylinitrosamine-induced HCC model, knockout miR-484 inhibited HCC tumorigenesis and decrease of tumor size. Signal pathway studies showed that aberrant IFNα/β initialized miR-484 expression. MiR-484 in turns blocked smad9, which results in accumulation of phosphorylated smad2 that drove cell transformation and HCC lesion formation [45].

Oxidative stress is another important reason for the HCC tumorigenesis and development by direct DNA damage [41]. Hepatitis B/C viruses increased reactive oxygen species (ROS) in hepatocytes and damage the GSH redox system. Excessive ROS impairs the DNA repair system, including the histone modification system and increases 8-Oxo-7,8-dihydroguanine (8-oxodG), a biomarker of DNA damage in hepatocytes. These changes increase HCC tumorigenesis and metastasis potential [26,46]. In contrast, TSA decreases mitochondrial ROS in HCC, increases histone H3 deacetylation and up-regulation of CYP2E1, a gene that induces apoptosis of HCC cells [47]. Redox homeostasis and epigenetics abnormalities are also observed in non-alcoholic fatty liver patients. This abnormal oxidative stress drives simple steatosis to non-alcoholic steatohepatitis. ROS also interferes with chromatin remodeling [48]. In alcoholic HCC patients, ROS contributes to target gene DNA methylation, histone alterations, and miRNA abnormalities [49].

### Impairing DNA repair and histone modification function increases the susceptibility of hepatocytes to developing HCC

In addition to DNA damages, impaired DNA repairing mechanism, including unbalance-epigenetic repairing mechanism, also caused hepatocarcinogenesis. Early study showed that many genomic and epigenetic abnormalities have been found in p53, β-catenin, p16CDK1, and DNA mismatch repair genes. However, no specific abnormal genetic or epigenetic changes for HCC have been found [50]. Recently, studies demonstrate that inactivation of a DNA repair gene, O^6^-methylguanine-DNA methyltransferase (MGMT) by hypermethylation in its promoter has close relationship with aflatoxin B1-mediated P53 mutation in HCC [51]. Analysis of clinical samples and cellular models have shown that MGMT in HCC was down-regulated by epigenetic mechanisms, such as MGMT promoter hypermethylation [52,53]. However, the details of this regulatory mechanism of CpG methylation of MGMT in HCC remains unclear.

Aberrant regulation of miR200 family by epigenetic mechanism is also linked to hepatocellular carcinogenesis. DNA methyltransferase I (DNMT1) and another histone methyltransferase, PcG protein enhancer of zeste homolog 2 (EZH2) bind with miR200a/b/429 promoter region and inhibit miR200a/b/429 expression in HCC and glioma cells. Silencing of EZH2 decreased DNMT1 binding on the miR200a/b/429 promoter and increased of miR200a/b/429 expression. These results indicated that EZH2 and DNMT1 silencing contributed to the progress of HCC or glioblastoma by regulation of miR200a/b/429 at epigenetic level [54].

Oxidative stress induced by various factors such as chemicals, virus, or irradiation also plays important role in epigenetic alteration and role in HCC development. ROS-mediated epigenetic instability results in two types of DNA alteration: (1) ROS induces the transcriptional factor, snail expression. Snail induces and recruits DNMT and HDACs to the TSG promoter, leads to TSG expression inhibition [55,56]. A ROS by-product, 8-Oxo-7,8-dihydroguanine (8-OxodG) recruits polycomb complex (PcG), such as DNMTs, SIRTs, ENH1 to the damage chromatin, which also inhibit TSG expression [57]. (2) On the other hand, the thymine is replaced by an unmethylated cytosine by base excision repair process. This results in TSG non-promoter region hypomethylation, chromatin instability which results in oncogene expression in HCC [58–60].

## Clinical implication

Histone H3 modifications directly or indirectly regulate HCC oncogene expression. These regulatory properties for HCC oncogenes have been successfully adopted for clinical purposes. Application of SAHA with 5-aza-dC together has a significant synergic effect on SAHA-mediate antiproliferation of HCC [61]. Pan-DACi panobinostat, an inhibitor of DNMT (DNA methyltransferases, DNMTs) demonstrates inhibition of promoter methylation of tumor suppressor genes like RASSF1A or APC and further inhibits HCC growth and proliferation [62]. HDAC inhibitor, vorinostatin also shows a synergic anticancer effect with mTOR inhibitor, sirolimus on the clinical trial [63]. The anti-VEGFR inhibitor resistant human HCC is related to an aberrant expression of G-actin monomer binding protein thymosin β4 (Tβ4). Sorafenib, VEGFR inhibitor, fails to improve the survival in the HCC patients with high Tβ4 expression level. Translational studies indicate that Tβ4 expression is initialized by epigenetic alterations and it is believed that it also contributes to the development of resistance to antiangiogenic therapy [64] (Table 1).

## Summary

Although more attention has been paid on epigenetic regulation of the HCC, the mechanism and clinical significance are still unclear. For example, in addition to histone H3, the importance of other histones like H4 or H1 and H2 are largely unknown. Even in histone H3, many critical amino acids, such as serines, lysine 14, 57 are relatively poor understand with regards to their role in HCC development. The effects of other modifications, such as phosphorylation, ubiquitination are also largely unknown.

Taken together, histone3 lysine modification is involved in carcinoma genes initialization, metabolism, angiogenesis, and metastasis. Altering these Histone3 lysine modifications significantly change HCC tumorigenesis properties and outcomes. These novel studies have been adopted in clinical trials and could become promising therapies for the HCC patients in the near future.

## Abbreviations

BA: bile acid
ChREBP: carbohydrate responsive-element binding protein
FASN: fatty acids synthase
FXR: farnesoid X receptor
HCC: hepatocellular carcinoma
HDAC: histone deacetylase
LncRNA: long non-coding RNA
MLL: mixed-lineage leukemia
PTEN: phosphatase and tensin homolog
ROC: reactive oxygen species
SAHA: suberoylanilide hydroxaminic acid
SRE: sterol regulatory element
VASH 2: vasohibin 2
